# *Lactobacillus acidophilus* and *L. plantarum* improve health status, modulate gut microbiota and innate immune response of marron (*Cherax cainii*)

**DOI:** 10.1038/s41598-020-62655-y

**Published:** 2020-04-03

**Authors:** Md Javed Foysal, Ravi Fotedar, Muhammad A. B. Siddik, Alfred Tay

**Affiliations:** 1 School of Molecular and Life Sciences, Curtin University, Bentley, WA Australia; 2 Department of Genetic Engineering and Biotechnology, Shahjalal University of Science and Technology, Sylhet, Bangladesh; 3 Department of Fisheries Biology and Genetics, Patuakhali Science and Technology University, Patuakhali, Bangladesh; 4 Helicobacter Research Laboratory, Marshall Centre for Infectious Disease Research and Training, School of Biomedical Sciences, University of Western Australia, Perth, WA Australia

**Keywords:** Bioinformatics, Reverse transcription polymerase chain reaction, Scanning electron microscopy, Microbiome

## Abstract

This study aimed to investigate the combined effects of two most potent probiotic bacteria *Lactobacillus acidophilus* and *Lactobacillus plantarum* on overall health and immune status of freshwater crayfish, marron under laboratory conditions. A total of 36 marron were distributed into six different tanks and two different feeding groups, control and probiotic-fed group. After acclimation, control group was fed with basal diet while probiotic group was fed 10^9^ CFU/mL per kg of bacterial supplemented feed for 60 days. The results showed no significant differences in weight gain, however, probiotic feed significantly enhanced some hemolymph parameters and biochemical composition of tail muscle. Histology data revealed better hepatopancreas health and higher microvilli counts in the marron gut fed probiotic diet. The probiotic bacteria triggered significant shift of microbial communities at different taxa level, mostly those reported as beneficial for crayfish. The probiotic diet also enriched the metabolic functions and genes associated with innate immune response of crayfish. Further correlation analysis revealed significant association of some taxa with increased activity for hemolymph and immune genes. Therefore, dietary *Lactobacillus* supplementation can modulate the overall health and immunity as well as gut microbial composition and interaction network between gut microbiota and immune system in crayfish.

## Introduction

Aquaculture has become an important food sector for meeting the overall protein demand for growing population. The global consumption of crustaceans has increased greatly and especially those with live transport abilities are increasingly becoming popular^1–3^. Marron (*Cherax cainni*) is one of the largest freshwater crayfish farmed in Western Australia (WA) that has high nutritive value and widespread consumer preferences^4,5^. In addition, long distance live transportation ability of marron further increases its international demand, and thus has become an ideal crayfish species for commercial farming^6,7^. However, the production of marron in WA has remained stagnant for a long time^7^. Selection of proper diets and maintaining the optimum water quality are the two most crucial factors required for marron farming^5,7^. Although no disease outbreaks have yet been reported, the interest and intend of intensification in marron farming can expose marron to possible crayfish pathogens including *Vibrio*, *Aeromonas* and *Rhodobacter*^5,6^. In the past, several laboratory based trials have been conducted^3,6–9^, however, finding a suitable diet and identifying the beneficial bacteria that potentially can influence the growth and immune performance of marron has still remained unknown.

Towards sustainable development of aquaculture, the use of feed additives including probiotics, prebiotics, synbiotics, parabiotics and phytogenics in crustacean’s diet to boost biological indices has gained extensive attention from the researchers and farmers. Probiotics are microorganisms associated with health and immune benefits for the host when administered in adequate amounts or numbers^10^. Among the probiotic bacteria, lactic acid bacteria (LAB) are considered as the most promising candidates for boosting the growth, gut health, immune defence mechanism against pathogenic bacteria^11–13^. *L. acidophilus* and *L. plantarum* are the two major bacterial species of LAB used as probiotics in aquaculture^12^. Improving growth and immune performance with dietary incorporation of *Lactobacillus acidophilus* has been reported in Nile tilapia (*Oreochromis niloticus*)^14,15^, rainbow trout (*Oncorhynchus mykiss*)^16^, common carp (*Cyprinus carpio*)^17^, stripped catfish (*Pangasianodon hypophthalmus*)^18^ and black swordtail (*Xiphophorus helleri*)^19^ as well as in crustaceans; white shrimp (*Litopenaeus vannamei*)^20^ and tiger shrimp (*Penaeus monodon*)^21^. *L. plantarum* is another important probiotic species that is known to produce various active compound like plantaricin with outstanding ability to counteract toxicity caused by various aquatic pathogenic bacterial species^22^. *L. plantarum* has shown potential to be used as probiotic in Nile tilapia (*O. niloticus*)^23^, common carp (*C. carpio*)^24,25^, rainbow trout (*O. mykiss*)^26^, silver pomfret (*Pampus argenteus*)^27^, African hybrid catfish (*Clarias gariepinus* Male × *Clarias macrocephalus* Female)^28^, and also in crustaceans including narrow clawed crayfish (*Astacus leptodactylus*)^29^, white shrimp (*L. vannamei*)^30,31^ and giant fresh water prawn (*Macrobrachium rosenbergii*)^32^. Studies also reported that combination of two or more probiotic bacteria including species from *Lactobacillus* can induce higher growth and immune performance of the host aquatic animals^20,33^. However, despite the beneficial role of LAB in finfish and crustaceans, their effects on overall health and immune status of marron is still unknown.

Recent development in “omics” technologies has enabled in-depth analysis of feeding effects on health and immunity of fish and crayfish^9^. In addition, advancement in information technology, data analysis packages and repository system allows to correlate various data from several trials and make interpretation easier and comprehensive^34^. This form of integrated data analysis packages can be used to explore the contributions of feed additives on growth performance, gut microbiota, innate immune response and disease resistance of crustaceans^35,36^. The aim of the present study was, therefore to investigate the impacts of dietary *L. acidophilus* and *L. plantarum* on health status, hemolymph parameters, intestine morphology and microbiota and innate immune responses of marron.

## Results

### Growth and health parameters

At the end of 60 days of feeding trial, growth was not significantly different between probiotic fed marron and the control. The probiotic fed marron did efficiently (*p* < 0.05) utilise the feed. The THC was positively influenced (*p* < 0.05) by the probiotic diet while no impacts on osmolality and lysozyme were recoded. The probiotic diet significantly (*p* < 0.05) improve the tail muscle crude protein and gross energy while the crude lipid content remained unchanged (Table 1).

**Table 1:** Health parameters of marron (*Cherax cainii*) after 60 days of feeding trial.

Parameters	Control	Probiotic	*p*-value
Weight gain (g)	6.55 ± 0.41	7.35 ± 0.55	0.059
Specific growth rate (%/day)	0.69 ± 0.04	0.86 ± 0.04	0.001
Feed conversion ratio	4.36 ± 0.17	3.83 ± 0.07	0.045
Muscle crude protein (%)	85.40 ± 0.75	88.2 ± 0.46	0.007
Muscle crude fat (%)	8.50 ± 0.19	8.40 ± 0.11	0.278
Muscle gross energy (MJ/kg)	20.44 ± 0.21	20.84 ± 0.09	0.029
Hemolymph lysozyme (unit/ml)	0.48 ± 0.02	0.51 ± 0.02	0.184
Haemolymph osmolality (mOsm/kg)	406.3 ± 2.65	408.8 ± 3.61	0.228
Total haemocyte count (million/ml)	8.40 ± 0.23	10.20 ± 0.64	0.002

### Gut microvilli and hepatopancreas structure

**Figure 1:**
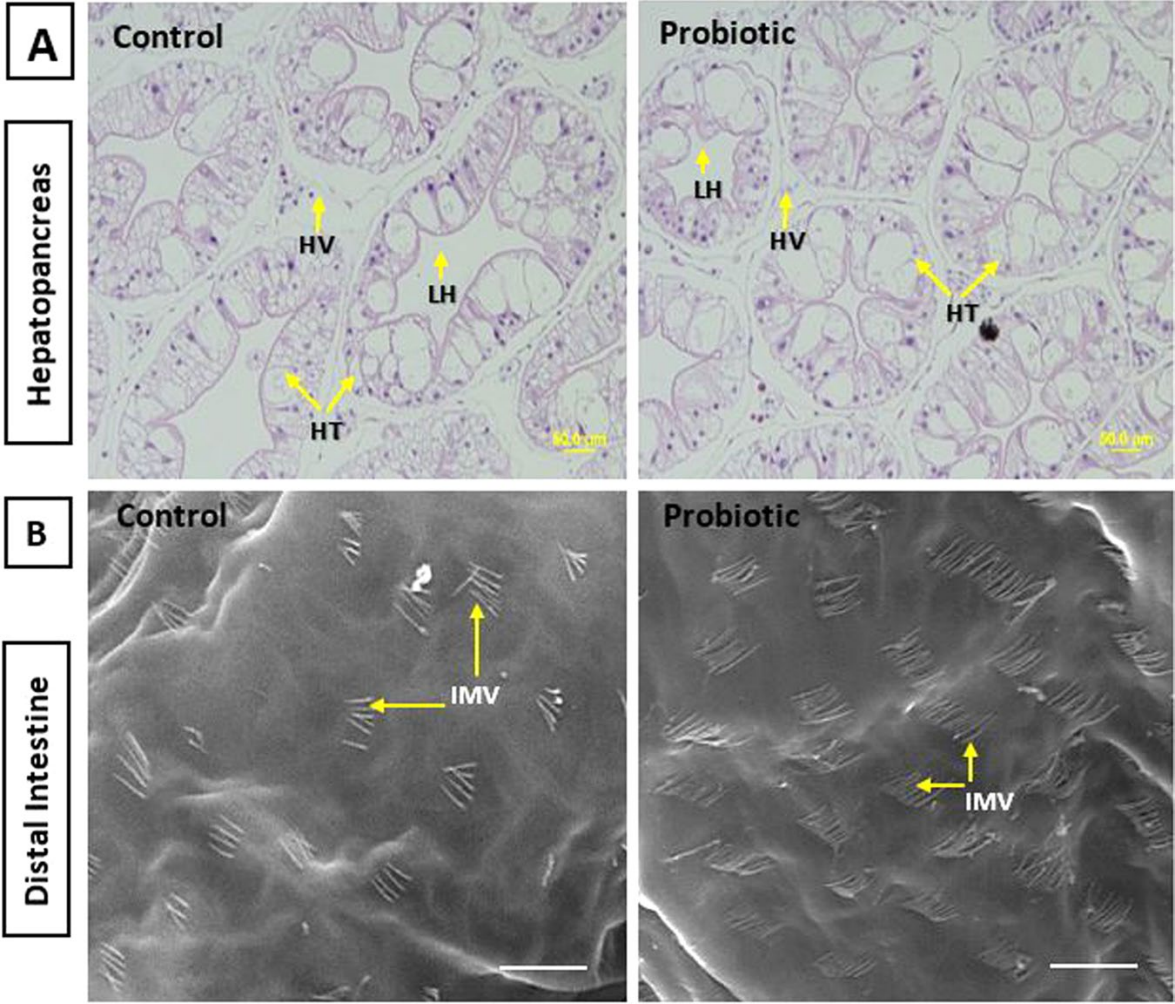
(**A**) Hepatopancreatine and intestinal morphology of freshwater crayfish, *Cherax cainii* fed control and probiotic diets for 8 weeks. Images are arbitrarily chosen from the micrograph observed in control and probiotic fed marron. Transverse section of hepatopancreas tubules showing reduced hepatopancreatic lumen and vacuole in probiotic fed group (H&E stain at 40×, scale bar = 50 µm). (**B**) High magnification (x50,000) electron micrograph showing increased number of microvilli in the distal gut of marron fed probiotic diet (scale bar = 20 μm). (HT: hepatopancreatic tubule, HV: hepatocyte vacuole, LHT: lumen of hepatopancreatic tubule, IMV: intestinal microvilli).

Histologically, healthy and balanced structure of hepatopancreas were found in the probiotic fed marron characterized by normal hexagonal hepatocytes and rare cytoplasmic vacuolization. Also, the lumen of hepatopancreatic tubule and hepatocyte vacuole were found comparatively smaller in probiotic fed group when compared to control (Fig. 1A). SEM analysis showed that the probiotic diet enhanced the number and density of microvilli in the distal gut of marron (Fig. 1B).

### Alpha-beta diversity of gut microbiota

**Figure 2:**
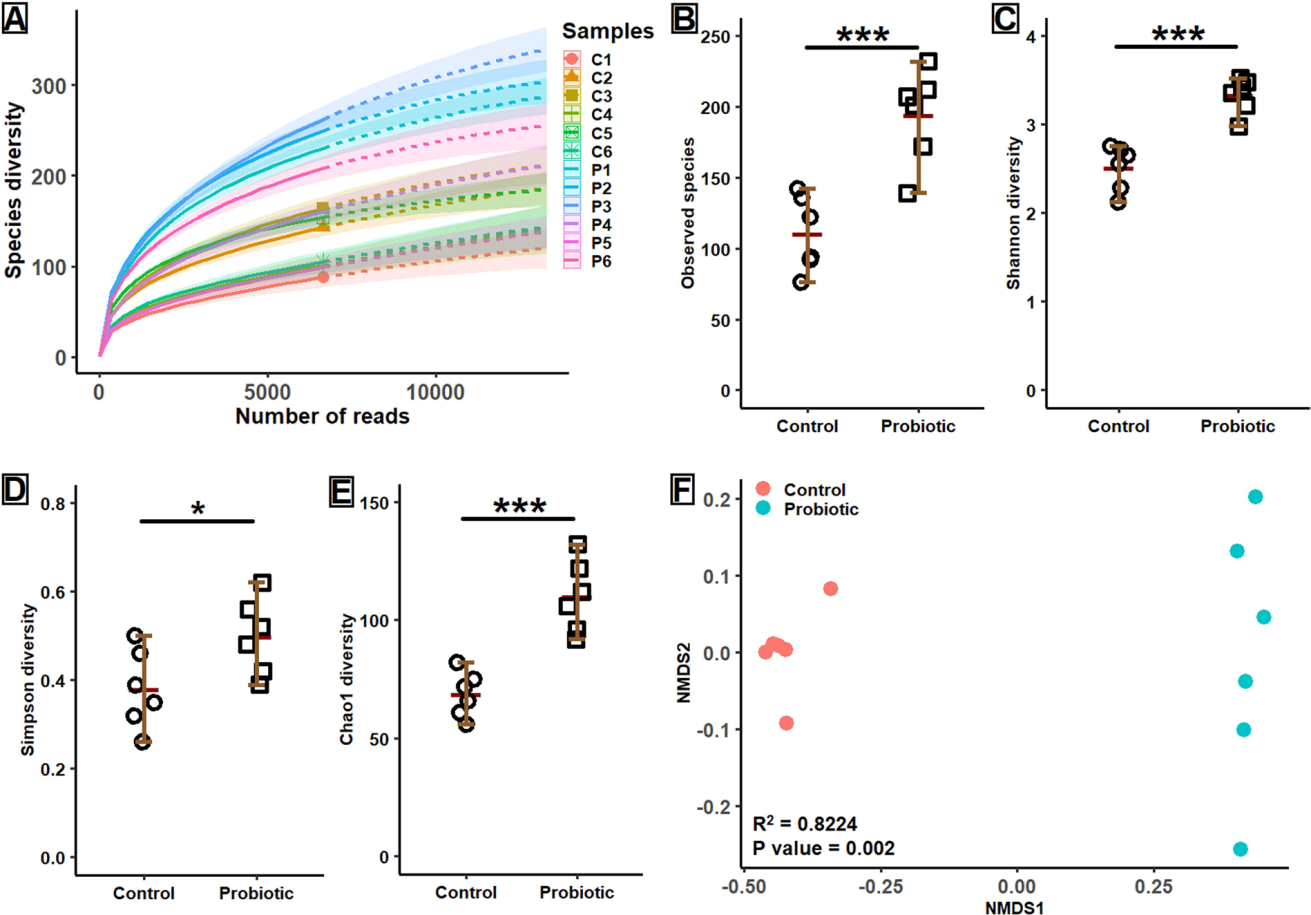
Alpha-beta diversity measurements of gut microbiota. (**A**) Rarefaction curve presenting the saturation level of sequencing in terms of observed species (**B–E**) Alpha diversity measurements in terms of observed species, Shannon, Simpson and Chao1 diversity indices. (**F**) Non-metric multidimensional scaling (NMDS) plot showing the clustering of samples based on Bray-Curtis dissimilarly of relative abundance. *Significant at α-level of 0.05. ***Significant at α-level of 0.001.

After filtering, 12 samples generated 804,713 quality reads that were classified into 984 OTUs, 16 phyla and 182 genera. The rarefaction curve indicated that each sample was sequenced at a higher depth and nearing about saturation to capture enough diversity (Fig. 2A). The rarefaction curve also showed that samples from probiotic fed marron had higher bacterial population than the control group. Further analysis revealed a significant increase (*p* < 0.001) of alpha diversity in terms of observed species, Shannon, Simpson and Chao1 diversity indices in the probiotic fed marron gut (Fig. 2B–E). A NMDS plot based on the relative abundance of bacterial OTUs and Bray-Curtis dissimilarities is shown in Fig. 2F. An R^2^ value of 0.82238 and *p value* of 0.002 revealed significant beta dispersion of bacterial communities and distances between the control and probiotic fed marron gut.

### Differential abundance of microbial communities

**Figure 3:**
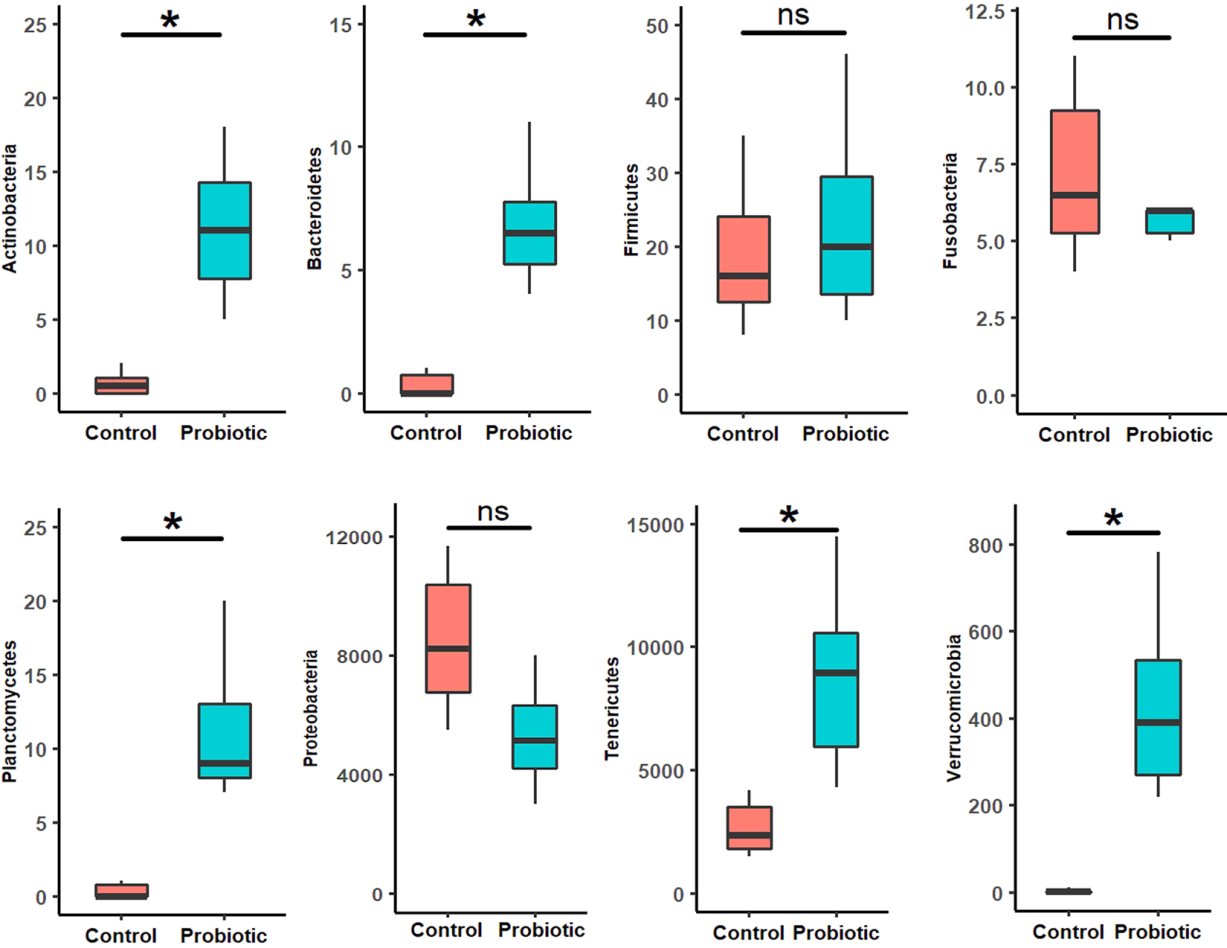
Differential abundance (*p* < 0.05) of bacterial communities in control and probiotic fed groups at phylum level.

**Figure 4:**
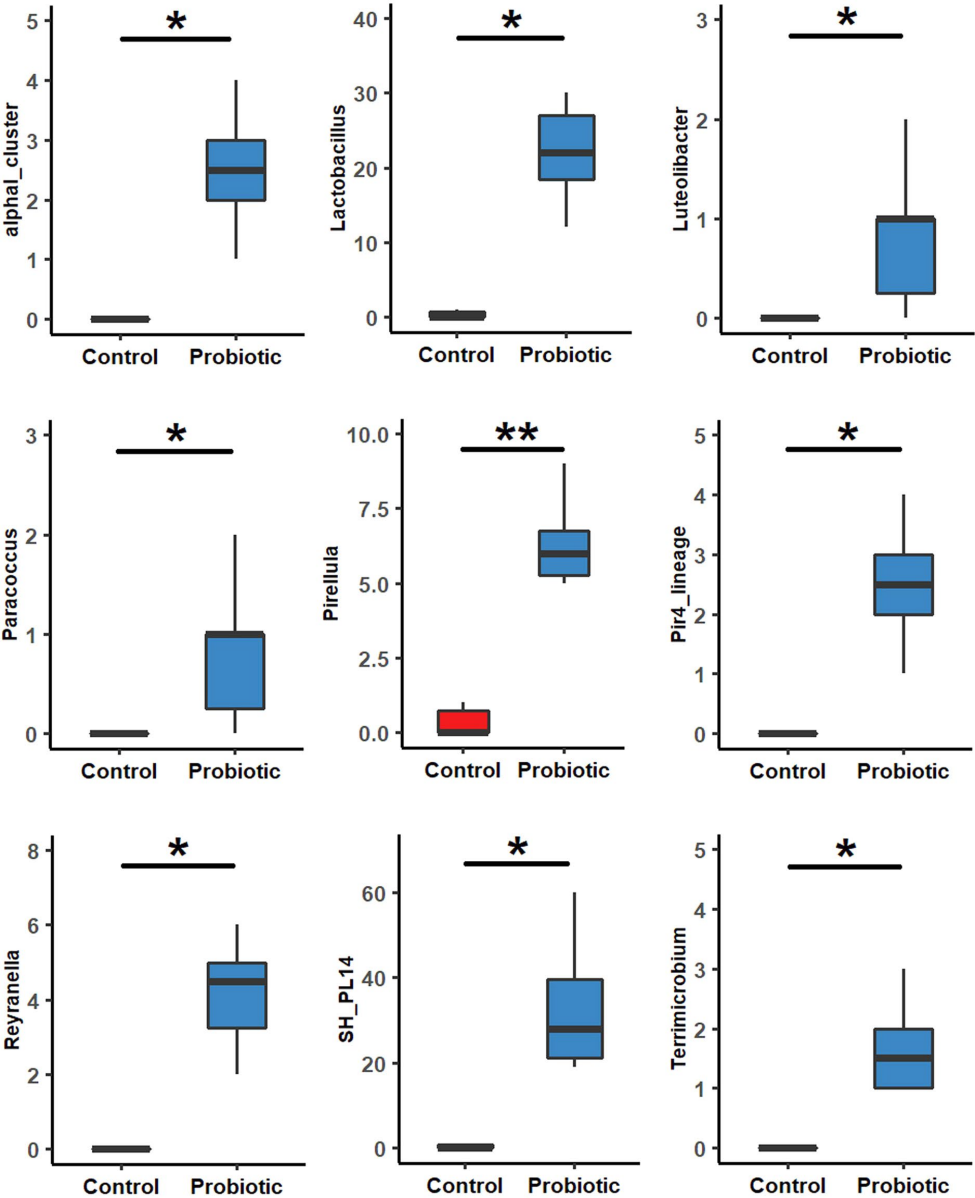
Differential abundance (*p* < 0.05) of bacterial communities in two different feeding groups at genus level.

**Figure 5:**
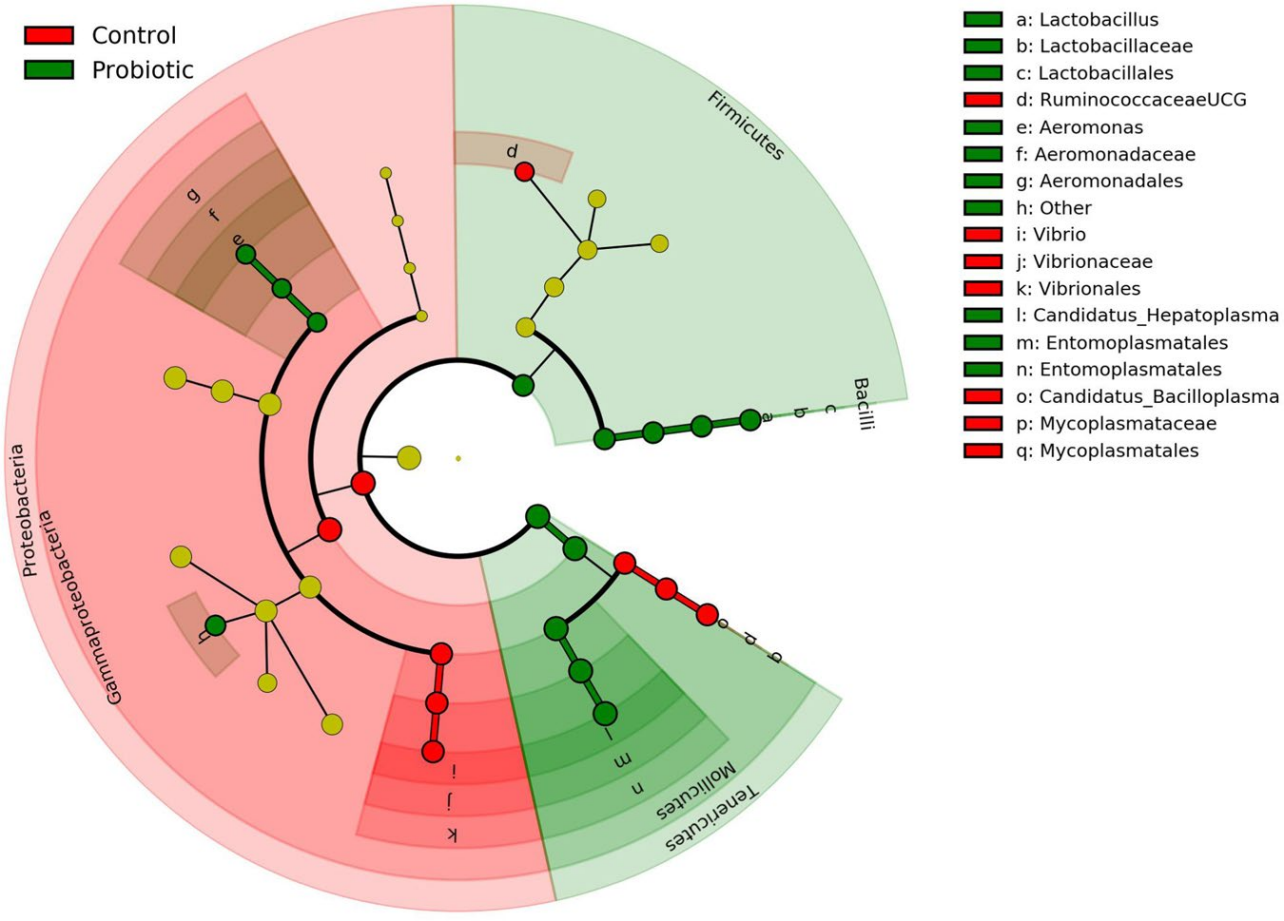
Cladogram representing the indicator bacteria at different taxa level in control and probiotic groups with LDA score ≥ 2.0 and at 0.05 level of significance.

At phylum level, Proteobacteria (87.7%) was the most dominant (*p* < 0.05) in the control group, followed by Tenericutes (11.12%) and Firmicutes (1.1%). While in the probiotic fed group, the relative abundance for Tenericutes (14.1%) and Bacteroidetes (1.2%) was significantly (*p* < 0.05) higher along with Actinobacteria, Planctomycetes and Verrucomicrobia (Fig. 3, Supplementary Table 1). Use of non-parametric *t-test* at 0.05 level of significance identified 9 genera including *Lactobacillus* that significantly were enriched in the probiotic fed marron. The other genera were AlphaI cluster, *Luteolibacter*, *Paracocccus*, Pir4 lineage, *Pirellula*, *Reyranella*, *Planctomyces* sp. SH-PL14 and *Terrimicrobium* (Fig. 4). Further analysis using LEfSe identified 17 taxa that were differentially expressed (p < 0.05) in two different groups. Among these, 10 including phyla Teniricutes, Firmicutes, class Bacilli, and genus *Candidatus* Hepatoplasma and *Lactobacillus* were significantly enriched in probiotic group while Proteobacteria (phylum) and *Vibrio* (genus) were the dominant bacteria in the control group (Fig. 5).

### Metabolic pathways and genes associated with immunity

**Figure 6:**
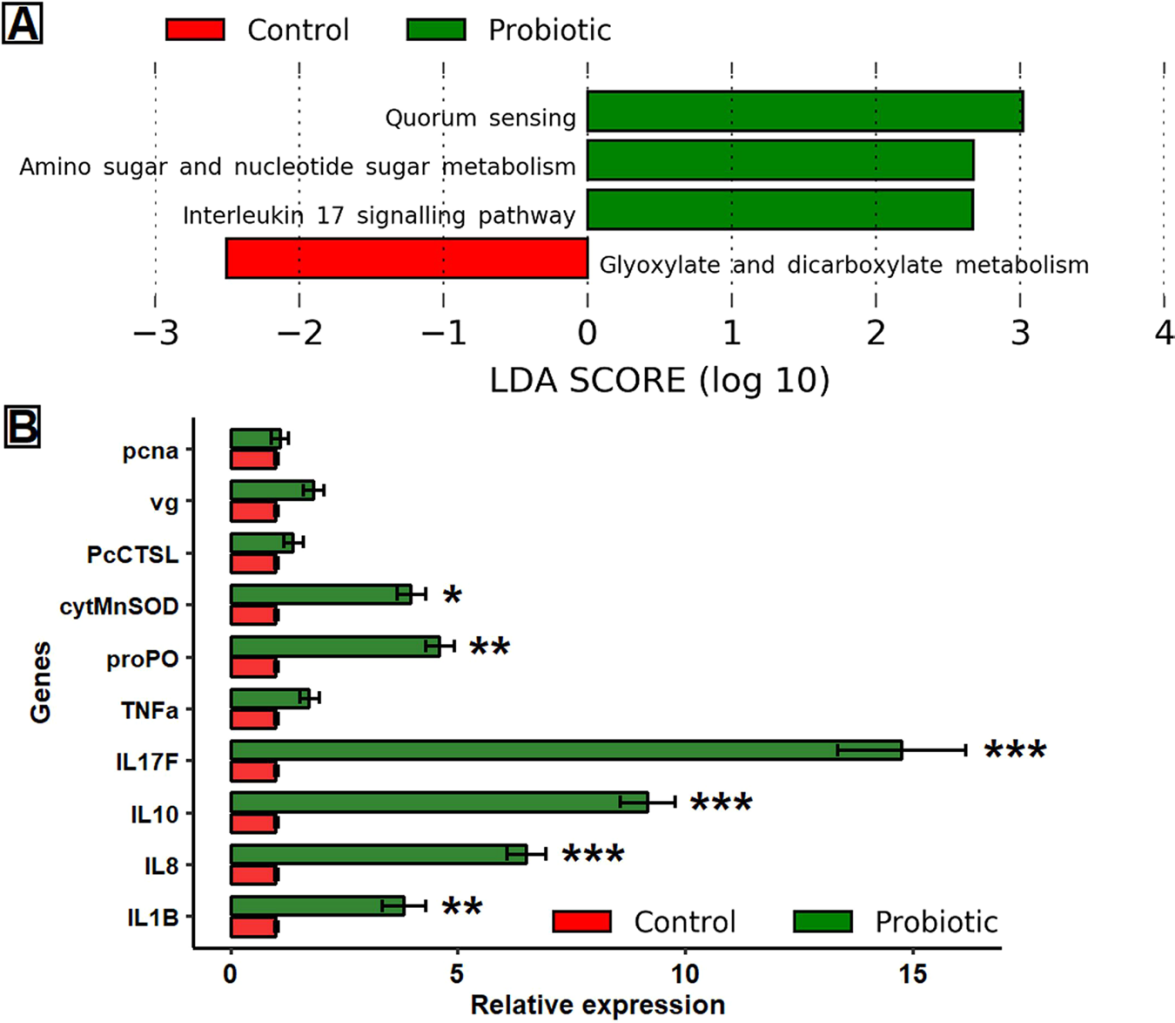
(**A**) Differentially abundant metabolic pathways based on 16S rRNA data in control and probiotic fed marron after 60 days of trial. The data extracted from Piphillin pipeline using KEGG database were compared using linear discriminant analysis (LDA) at strict LDA cut-off value of 2.0 and above. (**B**) Relative expression level (mean ± SE) of cytokines and crustacean genes associated with innate immune response of marron. *Significant at α-level of 0.05; **Significant at α-level of 0.005; ***Significant at α-level of 0.001.

The predicted metabolic pathways from 16S rRNA data using Piphillin and KEGG database showed that probiotics significantly influenced the enrichment of pathways related to amino sugar and nucleotide sugar metabolism, interleukin 17 signalling pathway and quorum sensing, whereas, glyoxylate and dicarboxylate metabolism was influenced in the absence of probiotics (Fig. 6A). The results of qRT-PCR from intestine tissue showed significant up-regulation of cytokine gene families (IL1β, IL8, IL10 and IL17F), proPO, and cytMnSOD in the probiotic fed marron. However, significant effects (*p* < 0.001) were observed for IL1β (3.8 fold), IL10 (9.2 fold) and IL17F (14.8 fold), followed by (*p* < 0.005) IL8 (6.5 fold) and proPO (4.6 fold), respectively. Significant (*p* < 0.05) upregulation was also observed for cytMnSOD (4.0 fold) while the expression level was static (*p* > 0.05) for TNF-α (1.7 fold), vg (1.8 fold), pcna (1.1 fold) and PcCTSL (1.4 fold) in the probiotic fed marron (Fig. 6B).

### The role of microbial communities in health and immunity

**Figure 7:**
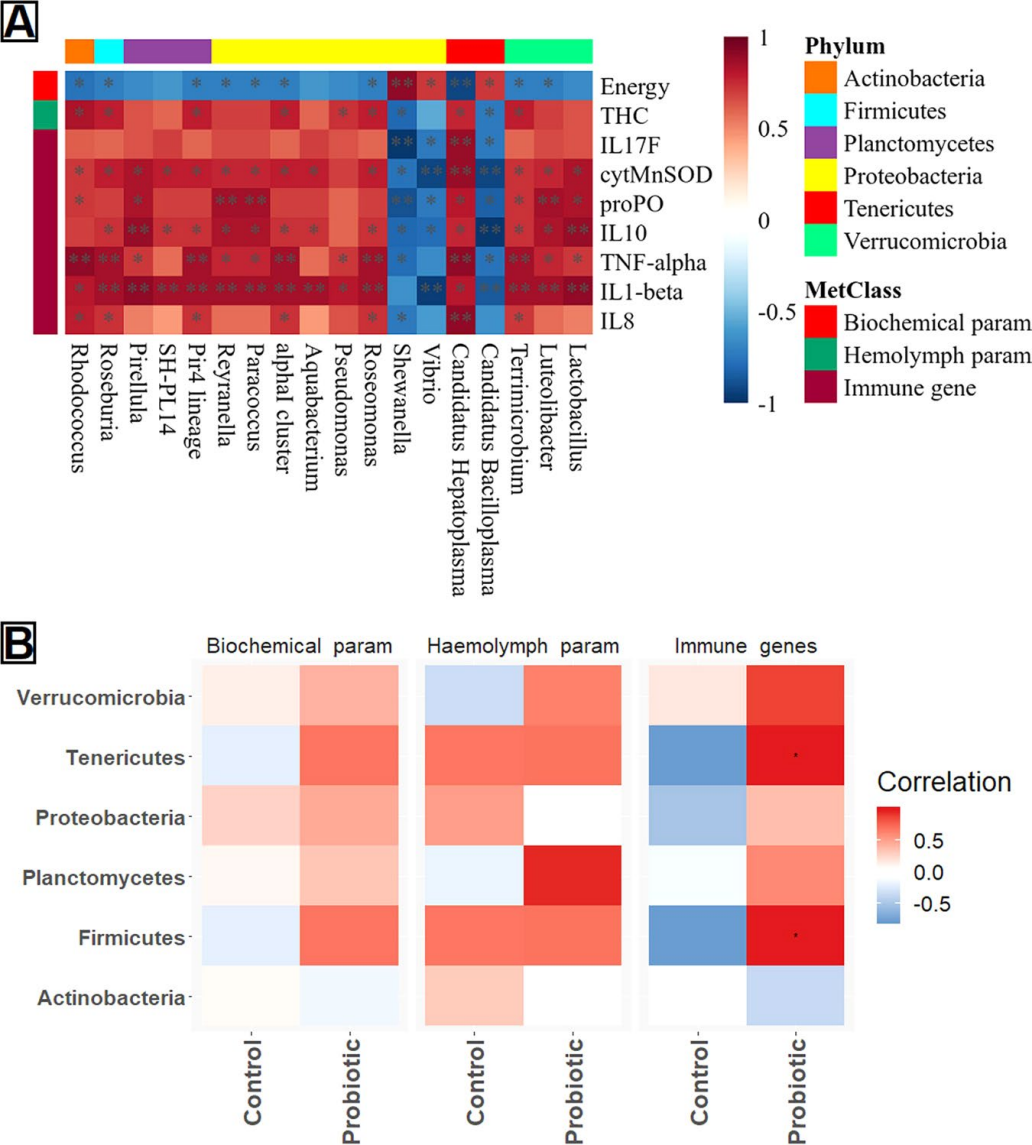
(**A**) A “heat map” showing Spearman correlation between microbial abundance and health and immune indices of marron. *Significant at α-level of 0.05; **Significant at α-level of 0.005. (**B**) Correlation “heat map” displaying the interactions between bacterial groups at phylum level and biological indices of marron after trial. Red representing positive interactions while green demonstrating negative interactions.

Pairwise “Spearman” correlation analysis among the microbial abundance and metadata, including health and immune indices showed a significant positive correlation between enriched bacteria at different taxa levels and immune indices of marron. Most of the enriched bacteria in the probiotic fed marron including *Lactobacillus*, *Candidatus* Hepatoplasma, *Terrimicrobium, Pirellula*, *Reyranella* and *Luteolibacter* were found strongly correlated to up-regulation of immune genes (Fig. 7A). *Vibrio*, *Shewanella* and *Candidatus* Bacilloplasma were found negatively correlated to immune response while positively correlated to gross energy in the tail muscle. The correlation network also revealed strong positive relationships between Firmicutes and Teniricutes with immune gene expression while hemolymph parameters correlated to Plactomycetes (Fig. 7B). In particular, *Candidatus* Hepatoplasma was strongly associated with IL17F and cytMnSOD upregulation, *Lactobacillus* and *Pirellula* with IL10 expression, *Reyranella* amd *Luteolibacter* with prePO up-regulation in the probiotic fed marron (Supplementary Table 2).

## Discussion

Dietary supplementation of feed additives have significant impacts on growth, immunity and disease resistance of crustaceans^3,7,9,29,37–40^. Probiotic in aqua diets, has positive influence on the growth and immune performance of commercially farmed shrimp and crayfish species. Among the probiotic bacteria, *Lactobacillus* species are widely used for their beneficial role in health and immunity of crayfish, *Astacus leptodactylus* and *C. cainii*^29,41^. Present study evaluated the combined effects of two most potent LAB on growth, hemolymph parameters, biochemical compositions of tail muscle, intestine and hepatopancreas structure, gut microbiota and immune genes of freshwater crayfish marron. Additionally, with the aid of bioinformatics, we investigated the correlations between microbial abundance and other metadata associated with growth and immunity. Therefore, this is a comprehensive study that combined biochemical, immunological, molecular and bioinformatic approaches to analyse the probiotic effects on overall health performance of marron. Although the growth data showed non-significant effects of probiotic diet, however, a *p value* of 0.059 suggest that higher growth performance could be achieved by this probiotic combinations, under a trial involving a longer feeding duration. Nevertheless, the growth data obtained with probiotic diet are really promising in considering the slow growing nature of marron under commercial farming conditions and when none of the previous studies found significant growth improvement with any of the diet formulations^7–9^. The augmented THC counts in hemolymph, and enhanced crude protein and gross energy in the tail muscle further revealed the beneficial role of probiotic diet. These parameters are crucial for determining the health and immune status of crayfish including marron^6–8^.

Increasing evidence has found a close association between the intakes of diet-supplemented probiotic strains and gut health of fish and crustacean^9,42–44^. A proper orientation of gut microvilli, villi length, and healthy structure of hepatopancreas cell are associated with proper nutrient absorption and utilization, and thus higher growth and immune function^45^. The SEM and histology image revealed better morphology and orientation of gut and hepatopancreas in the probiotic fed marron than the control. The results is consistent and compatible with other previous studies that fed dietary supplementation of *Lactobacillus* bacteria including *L. plantarum*^46,47^. In crayfish, no published data are currently available, however, a previous study found that dietary supplementation of *Lactobacillus pentosus* in the commercial diet of shrimp protect the hepatopancreas from pathogen invasions^42^. Enferadi *et al*. reported a significant increase of intestine enzyme activity, and higher digestibility and absorption of feed in rainbow trout fed *L. plantarum* supplemented diet^16^. Therefore, better gut and hepatopancreas structure in the probiotic fed marron may be attributed to the beneficial effects of *Lactobacillus* bacteria on digestion, absorption and nutrient utilization.

Modulation of gut microbiota and their interactive networks results in production of several types of metabolites, vitamins and antimicrobial agents that affect largely on the host physiology and immune response^48–50^. The gut microbes of freshwater fish are commonly dominated by phyla Proteobacteria, Firmicutes, Bacteroidetes, Actinobacteria, and Fusobacteria^51,52^. Firmicutes, Bacteroidetes, and Teniricutes are defined as the beneficial bacteria of the gut and their positive role in growth performance, immunity, digestion and disease resistance of aquatic animals^53–56^. Additionally, they also play an influential role in augmentation of other beneficial bacteria in the gut^57–59^. The enriched bacteria at genus level in the probiotic fed marron can be correlated with better growth and immune performance and water quality from previous studies. For instances, *Pirellula* abundance was found correlated to essential amino acids such as isoleucine, leucine and valine production in grass carp (*Ctenopharyngodon idellus*) and blunt snout bream (*Megalobrama amblycephala*)^34^. Another genus *Reyranella* that is phylogenetically very close to Rhodospirillaceae, also reported to play a crucial role in nitrate reduction from soil and freshwater^60,61^. The bacteria recently been characterized from Tropical gar fish (*Atractosteus tropicus*) where its positive role on fish adaptation and survival has been described^62^. *Paracoccus* species identified from turbot (*Scophthalmus maximus*), European flounder (*Platichthys flesus*) and wild common dab (*Limanda limanda*) gut were found strongly correlated to high content of polyunsaturated fatty acids (PUFA), especially docosahexaenoic acid (DHA) and eicosapentaenoic acid (EPA) that have potential health benefit effects on aquatic animals as well as on humans, especially the role of PUFA in liver and brain function, inflammation, cardiovascular diseases, obesity and diabetes has been widely documented^63–65^. Another genus *Luteolibacter* is described to play a positive role in *Lactobacillus* colonization in the fish gut, however, the mechanism is not clearly stated yet^66,67^. The positive role of *Lactobacillus* in the gut, health and immune status, and disease resistance of aquatic animals has also been investigated^16,30,42,68,69^. In a most recent study, dietary supplementation of 10^9^ CFU/mL of *L. plantarum*, a same concentration to the present study significantly increased the haemolymph parameters, enzymatic activity and LAB counts in the gut of narrow clawed crayfish (*Astacus leptotylus*)^29^. Therefore, addition of *L. acidophilus* and *L. plantarum* in diet generated an augmented community of beneficial bacteria for aquatic animals that might be associated with improved health and immune status of marron in this study.

Unlike fish and other vertebrates, crustaceans lacks adaptive immune system and therefore solely rely on innate immune response generated primarily from the immunocompetent cells and mucus of intestinal mucosal membrane^9,70,71^. In the present study, we selected 10 genes that are reported to play a crucial role in innate immune response of crayfish earlier^7,9,72–74^. We found upregulated expression of pro-inflammatory cytokines, and crustacean’s hemolymph genes in the probiotic fed marron. To prevent inflammation related damages due to upregulated expression of pro-inflammatory cytokines, the anti-inflammatory cytokines (IL10) was also stimulated in the probiotic fed marron to neutralize inflammation. This upregulation was significant due to the pro and anti-inflammatory mechanisms, aquatic animals balanced the immune response for better defence against stress and infection^56^. Though the presence of cytokine family genes in crustacean tissue including crayfish are evident^41,75–79^, however, their role have not been studied yet. Hence, further transcriptomic analysis of mRNA is recommended to identify the expressed genes under cytokine family and its level of expression in the intestinal tissue of marron.

The predictive role of microbial communities and correlating them with the health and immune indices of marron is the most significant findings of this study. Firstly, the predicted enriched IL17 signalling pathway from 16S rRNA data has been validated by upregulation of IL17F gene in qPCR assay. We also investigated the correlations between microbial abundance and biological indices and found the significant role of Firmicutes, Bacteroidetes and Teniricutes in enhancing the innate immune response while Proteobacteria was mostly linked to health performance. The positive role of Fimicutes and *Lactobacillus* on innate immune response including cytokine expression, and Bacteroidetes, Teniricutes in expression of anti-microbial peptides (AMPs), gut health and immunity has been reported in northern snakehead (*Channa argus*)^56^ and in Chinese mitten crab (*Eriocheir sinensis*)^80^, respectively. However, although reported abundant in gut and considered as beneficial bacteria for the health of crustaceans^7,81–83^, the role of *Candidatus* species including *Candidatus* Bacilloplasma and *Candidatus* Hepatoplasma, have not been investigated yet. In this regard, its role in health and immunity of crayfish could be useful in future diet preparations, disease resistance and metabolomics studies, and thus need further investigations.

The above findings demonstrated that the supplementation of potential probiotic *L. acidophilus* and *L. plantarum* to diet, significantly promote the gut and hepatopancreas health, immune response as well as microbial composition and interaction network in marron. This work will help to understand the probiotic mechanism and possibility of using *L. acidophilus* and *L. plantarum* as potent probiotic bacterial combinations in marron aquaculture.

## Materials and methods

### Ethics statement

Formal ethics approval is not necessary for the laboratory trial with invertebrates at Curtin University. However, the study was performed according to the guidelines of Animal Welfare Act, Western Australia and the Australian Code for the Care and Use of Animals for Scientific Purposes (2014).

### Experimental set-up

A total of 36 marron (70.2 ± 0.6 g) were procured from Blue Ridge Marron Farm (Manjimup, Western Australia) and transported in live condition to Curtin Aquatic Research Laboratories (CARL) at technology park, Bentley, Western Australia. Marron rearing tanks at CARL were filled with 150 L underground freshwater a week before experimental trial. Marron were then distributed into six different tanks with a density of six marron per tank and two different dietary treatment groups, control and probiotic. Constant temperature (22 °C) and constant aeration were maintained using submersible thermostat (Aqua One, Perth, Australia) and air diffusers (Aqua One, Perth, Australia), respectively. Marron were acclimated for 7 days before the commencement of the trial and both groups were served with standard basal diet during acclimation period.

### Bacterial culture, diet formulation and feeding

*L. acidophilus* and *L. plantarum* were purchased from Nature Way Probiotic (Warriewood, New South Wales, Australia) in powdered form. The bacteria were then cultured in MRS broth (Sigma-Aldrich, Germany) overnight at 37 °C. Bacterial cells were then harvested by centrifugation at 4000 rpm for 10 min, washed twice with phosphate buffer saline (PBS) followed by re-suspension in the same buffer. The colony forming units (CFU) of respective bacteria was calculated following standard serial dilution method. One hundred microliter of bacterial broth from each dilution was cultured in MRS agar (Sigma-Aldrich, Germany), incubated for 48 h at 37 °C under anaerobic conditions. Subsequently the dilution containing 10^9^ CFU/mL *L. acidophilus* and *L. plantarum* counts in culture plate was selected based on results of previous study on crayfish^29^. The ingredients of basal diet was purchased from a commercial feed supplier (Glenn Forest, Perth, Australia). The probiotic diets were formulated and prepared at CARL following previously described method^9,84^. Briefly, the ingredients were passed gently though 100 µm mesh sieve and rigorously homogenized to get uniform particle size. The suspension of *L. acidophilus* and *L. plantarum* in water were then added at 10^9^ CFU/mL per kg of feed using sprayer until the bacterial suspension for 1 kg diet was finished. Feeding pellet was prepared using a mince mixture followed by vacuum drying oven at 37 °C for overnight and then stored at 4 °C in air tight jars before the use. The proximate composition of final diet (Supplementary Table 3) was determined according to the method of Association of Official Analytical Chemists, AOAC^85^ and bacterial CFU was calculated on MRS agar plate. Throughout the trial, marron were fed based on their satiation level, once every day at 6 PM for 60 days at a rate of 1% of total biomass per tank^8^. Control group fed basal diet while probiotic group served with *Lactobacillus* supplemented diet.

### Sampling

For analysis of haemolymph parameters, health and immune indices, 12 marron, two randomly selected from each tank were used. For DNA extraction and microbiome analysis, total 24 marron, four randomly selected marron from each tank were selected. The hindgut content of two randomly selected marron from each tank were homogenized and pooled together, eventually prepared two pools of sample from each tank and six for each treatment. Finally, for gene expression analysis, the whole intestine of two randomly selected marron from each tank (n = 12) was used for RNA extraction.

### Growth parameters

At the end of the experimental trial, the marron growth performance was calculated by using the following formulae:

$$\begin{array}{rcl}{\rm{Weight}}\,{\rm{gain}}\,({\rm{WG}},{\rm{g}}/{\rm{marron}}) & = & \left[\frac{{\rm{mean}}\,{\rm{final}}\,{\rm{body}}\,{\rm{weight}}-{\rm{mean}}\,{\rm{initial}}\,{\rm{body}}\,{\rm{weight}}}{{\rm{mean}}\,{\rm{initial}}\,{\rm{body}}\,{\rm{weight}}}\right]\\ {\rm{Specific}}\,{\rm{growth}}\,{\rm{rate}}\,({\rm{SGR}}, \% /{\rm{day}}) & = & \left[\frac{\mathrm{ln}\,({\rm{final}}\,{\rm{body}}\,{\rm{weight}})-\,\mathrm{ln}\,({\rm{pooled}}\,{\rm{initial}}\,{\rm{body}}\,{\rm{weight}})}{{\rm{days}}}\right]\times 100\\ {\rm{Feed}}\,{\rm{intake}}\,({\rm{TFI}},{\rm{g}}) & = & \left[\frac{{\rm{dry}}\,{\rm{feed}}\,{\rm{consumed}}}{{\rm{number}}\,{\rm{of}}\,{\rm{marron}}}\right]\\ {\rm{Feed}}\,{\rm{conversion}}\,{\rm{ratio}}\,({\rm{FCR}}) & = & \left[\frac{{\rm{dry}}\,{\rm{feed}}\,{\rm{fed}}}{{\rm{wet}}\,{\rm{weight}}\,{\rm{gain}}}\right]\end{array}$$

### Haemolymph parameters

Hemolymph osmolality was measured following method described by Sang and Fotedar, 2004^86^. Briefly, 0.1 mL marron hemolymph was collected from the peritoneal cavity and mixed with 0.1 mL precooled anticoagulant (0.1% glutaraldehyde in 0.2 M sodium cacodylate, pH 7.0 ± 0.2) using 0.5 mL syringe. The osmolality of anticoagulant added hemolymph solution was measured using Cryoscopic Osmometer-Osmomet 030 (Gonotec, Berlin, Germany). Hemolymph lysozyme activity was measured using turbidimetric assay as described by Mai and Fotedar, 2018^87^. Fifty microliters of anticoagulant mixed hemolymph solutions were transferred to 96 well plate (Iwaki, Tokyo, Japan). Then, after 15 minutes of incubation at room temperature, 50 µL of PBS (0.25 mg/mL) suspended *Micrococcus lysodeiktikus* (Sigma-Aldrich, St. Louis, MO, USA) solution was added to the well plate. The absorbance of the well plate was measured at every minute for 5 min at 450 nm wavelength under MS212 reader (Titertek Plus, Tecan, Grodig, Austria)^9^. Finally for total haemocyte counts (THC), one drop of anticoagulant added hemolymph solution was taken onto microscope slide. The cells were counted under hemocytometer (Nauabuer, Germany) with 100X magnification and the THC was calculated following previously described standard method^88^.

### Biochemical composition of tail muscle

The biochemical composition of tail muscle including crude protein, crude fat and gross energy were measured following standard methods published by the Association of Official Analytical Chemists, AOAC international^85^. Tail muscle crude protein was calculated after following Kjeldahl method (N× 6.25) using sulfuric acid (H_2_SO_4_) and copper catalyst tablets in Kjeltec Auto 1030 analyzer (Foss Tecator, Höganäs, Sweden). The crude fat content (in percentage) in the tail muscle was calculated using Soxtec System HT6 (Tecator, Höganäs, Sweden). The gross energy in the tail muscle was calculated in bomb calorimeter (Heitersheim, Germany).

### Hepatopancreas and intestinal mucosal morphology

After 60 days of feeding trial, six randomly selected marron were selected from each treatment for histology of hepatopancreas and scanning electron microscopy of intestinal microvilli. Hepatopancreas samples were dehydrated in ethanol, equilibrated in xylene and embedded in paraffin wax following standard histological techniques. By using a rotary microtome, a section of approximately 5 µm in size was cut from each paraffin block and stained with Hematoxylin-Eosin (H&E) solution followed by histological examination under a light microscope (BX40F4, Olympus, Tokyo, Japan).

For SEM analysis, intestinal samples were prepared according to previously described standard method for marron with slight modifications^3^. Briefly, the dissected transverse segments (~1 mm long) of intestinal specimens were bathed immediately in 3% glutaraldehyde containing 0.1 M cacodylate buffer followed by overnight (24 h) incubation at 4 °C. Samples were then rinsed briefly with cacodylate buffer and PBS prior to secondary fixation using 1% OsO4, followed by three consecutive washes in deionized distilled water for 5 min, followed by dehydration in ethanol (50, 70, 95 and 100% at 250 W, 5 min each). The samples were dried by washing in a series of 50%, 75% and 100% (twice) hexamethyldisilizane (HMDS) in ethanol solutions for 5 min. The processed samples were then dried at room temperature and mounted on a stub using carbon tape, coated with gold and viewed under a pressure scanning electron microscope (SEM, model Phillips XL 30, FEI, Hillsboro, OR, USA). The inner part of the digestive tract was assessed under 5000 X magnification for distribution and densities of microvilli. The images acquired from SEM were used to calculate the number of hindgut microvilli by counting and averaging microvilli on each slide (n = 3) using digital imaging software (Adobe Photoshop CC 2015, Adobe System Incorporated, USA).

### Illumina sequencing

The bacterial DNA from pooled samples was extracted using DNeasy Blood and Tissue Kit (Qiagen, Hilden, Germany) following manufacturer’s instructions. After quantification in NanoDrop spectrophotometer (Thermo Fisher Scientific, Waltham, MA, USA), extracted DNA was diluted into 50 ng/μl final concentration for PCR. Fifty microliters of PCR master mix was prepared by mixing 25 µL Hot Start 2X Master Mix (New England BioLab Inc., Lawrenceville, GA, USA), 2 µL of respective template DNA, 1 µL of each V3 and V4 sequencing primers (Part # 15044223 Rev. B) and 21 µL of DEPC treated water (Sigma-Aldrich, Germany). Forty cycles of amplification reactions were then performed in a BioRad S100 Gradient Thermal Cycler (Bio-Rad Laboratories, Inc., Foster City, California, USA). After visualization of PCR products in 1% agarose gel and subsequent clean-up with beads, each PCR amplicon was barcoded via a secondary PCR according to the Illumina standard protocol (Part # 15044223 Rev. B). Each sample was then sequenced up to 40,000 reads on an Illumina MiSeq platforms (Illumina Inc., San Diego, California, USA) at Harry Perkins Institute of Medical Research, Western Australia, using a v3 kit (600 cycles, Part # MS-102-3003).

### Gene expression analysis

In this study, ten (10) genes (Supplementary Table 4) associated with innate immune response of crayfish were selected for expression analysis after trial^9,72–74^. The whole intestine tissue samples preserved at −80 °C in RNA *Later* solution (Sigma-Aldrich, Germany) were thawed, rinsed with DEPC treated water (Sigma-Aldrich, Germany), and homogenized in TissueLyser (Qiagen, Hilden, Germany). For prophenoloxidase (proPO) and cytosolic manganese superoxide dismutase (cytMnSOD), pellet from centrifuged haemolymph samples (in pre-cooled anticoagulant) was processed for RNA extraction according to method described by Liu *et al*., 2013^72^. RNA from whole intestine tissue and pellet samples was extracted using RNeasy Mini Plus Kit (Qiagen, Hilden, Germany) following manufacturer’s instructions. RNase free DNase-I (Qiagen, Hilden, Germany) was added during extraction for the removing of DNA associated impurities. The quality of extracted RNA was checked in 1% agarose gel and quantity was measured in NanoDrop spectrophotometer 2000c (Thermo Fisher Scientific, USA), respectively. The cDNA library was synthesized from 1 µg of RNA using Omnicript RT kit (Qiagen, Hilden, Germany). The quantitative real-time PCR (qRT-PCR) to analyse the mRNA expression level was performed using PowerUp^TM^ Cyber Green Master Mix (Thermo Scientific, USA) with 7500 Real-Time PCR System (Applied Biosystems, USA). Analysis of qRT-PCR data for relative expression was performed using the 2^−ΔΔCT^ method, after normalisation against the β-actin reference gene^89^.

### Bioinformatics

The initial quality of Illumina sequences was checked in FastQC pipelines^90^. Sickle program was used for quality trimming, and following trimming reads of <200 bp length and q < 20 quality were eliminated^91^. Merging of reads, quality checking, filtering of chimeric sequences, open-reference clustering of sequences into operational taxonomic units (OTUs) at 97% similarity threshold level and removing of singletons OTUs was performed in micca otu (version 1.7.0)^92^. Taxonomic classification of OTUs was performed against SILVA database at 97% similarity threshold level^93^. Multiple sequence alignment was performed in PASTA algorithm^94^. Rarefaction depth point was set at 13950 bp and subsequent measurement of alpha beta diversities were performed in QIIME pipeline (version 1.9.1)^95^ and different R packages. Briefly alpha diversity was calculated in terms of species richness and Shannon index using student independent *t-test*. Non-parametric statistical analysis of the distance metric was performed with 1000 permutations using ANOSIM. The beta diversity analysis was performed as nonmetric multidimensional scaling plot (NMDS) using permutational analysis of variance (PERMANOVA) based on Bray-Curtis dissimilarity matrix. Differential abundance at genus level, global similarity, pairwise microbiome-metadata correlations, multivariate regression were performed using LEfSe (Linear Discriminant Analysis Effect Size) and MMCA microbiome pipeline^96,97^. Differentially expressed metabolic pathways in two different groups based on 16S rRNA data were predicted using Piphillin (http://secondgenome.com/Piphillin) in support of KEGG database (May, 2017 release), BioCyc 21.0 and LEfSe^96,98^. In all cases, p value of <0.05 was considered as statistically significant.

## Supplementary information

Supplementary tables.

## Data Availability

The raw sequence data in FASTQ files are currently available at National Centre for Biotechnology Information (NCBI) BioProject under the accession number PRJNA579035.
