# Supplementary material for: Lactobacillus acidophilus and L. plantarum improve health status, modulate gut microbiota and innate immune response of marron (Cherax cainii)
Source: Sci Rep. 2020 Apr 3;10:5916. doi: 10.1038/s41598-020-62655-y (PMC7125160; doi:10.1038/s41598-020-62655-y)
Supplement: Supplementary file 1 — Supplementary tables. [file 41598_2020_62655_MOESM1_ESM.pdf]

**Lactobacillus acidophilus and L. plantarum improve health status, modulate gut microbiota and innate immune response of marron (Cherax cainii)**

Md Javed Foysal[1,2*], Ravi Fotedar[1], Muhammad A.B. Siddik[3], Alfred Tay[4]

[1] School of Molecular and Life Sciences, Curtin University, Bentley, WA, Australia

[2] Department of Genetic Engineering and Biotechnology, Shahjalal University of Science and Technology, Sylhet, Bangladesh

[3] Department of Fisheries Biology and Genetics, Patuakhali Science and Technology University, Patuakhali, Bangladesh

[4] Helicobacter Research Laboratory, Marshall Centre for Infectious Disease Research and Training, School of Biomedical Sciences, University of Western Australia, Perth, WA, Australia

[*]Corresponding author:

Email address: mjfoysal-geb@sust.edu or mdjaved.foysal@postgrad.curtin.edu.au

Telephone: +61 8 9266 4508: mobile +61451404337

ORCID: https://orcid.org/0000-0002-2064-8897

**Supplementary Table 1**. Mean ± SE of bacteria in two different fed groups at phylum level after trial

| Phylum | Control (%) | Probiotic (%) |
|---|---|---|
| Proteobacteria | 87.7 ± 4.16 | 84.54 ± 4.81 |
| Tenericutes | 11.1 ± 4.26 | 14.05 ± 4.68 |
| Bacteroidetes | 0.2 ± 0.01 | 1.2 ± 0.09 |
| Firmicutes | 1.1 ± 0.29 | 0.4 ± 0.02 |

**Supplementary Table 2.** Correlation between microbial communities and metadata of marron health and immunity

| Phylum | Genus | MetaClass | Meta | R | Pvalue |
|---|---|---|---|---|---|
| Actinobacteria | Rhodococcus | Biochemical param | Energy | -0.762 | 0.028 |
| Actinobacteria | Rhodococcus | Hemolymph param | THC | 0.833 | 0.01 |
| Actinobacteria | Rhodococcus | Immune gene | IL17F | 0.619 | 0.102 |
| Actinobacteria | Rhodococcus | Immune gene | cytMnSOD | 0.714 | 0.047 |
| Actinobacteria | Rhodococcus | Immune gene | proPO | 0.714 | 0.047 |
| Actinobacteria | Rhodococcus | Immune gene | IL10 | 0.69 | 0.058 |
| Actinobacteria | Rhodococcus | Immune gene | TNF-alpha | 0.905 | 2.00E-03 |
| Actinobacteria | Rhodococcus | Immune gene | IL1-beta | 0.81 | 0.015 |
| Actinobacteria | Rhodococcus | Immune gene | IL8 | 0.786 | 0.021 |
| Firmicutes | Roseburia | Biochemical param | Energy | -0.714 | 0.047 |
| Firmicutes | Roseburia | Hemolymph param | THC | 0.786 | 0.021 |
| Firmicutes | Roseburia | Immune gene | IL17F | 0.595 | 0.12 |
| Firmicutes | Roseburia | Immune gene | cytMnSOD | 0.786 | 0.021 |
| Firmicutes | Roseburia | Immune gene | proPO | 0.69 | 0.058 |
| Firmicutes | Roseburia | Immune gene | IL10 | 0.738 | 0.037 |
| Firmicutes | Roseburia | Immune gene | TNF-alpha | 0.857 | 6.50E-03 |
| Firmicutes | Roseburia | Immune gene | IL1-beta | 0.857 | 6.50E-03 |
| Firmicutes | Roseburia | Immune gene | IL8 | 0.738 | 0.037 |
| Planctomycetes | Pirellula | Biochemical param | Energy | -0.667 | 0.071 |
| Planctomycetes | Pirellula | Hemolymph param | THC | 0.643 | 0.086 |
| Planctomycetes | Pirellula | Immune gene | IL17F | 0.643 | 0.086 |
| Planctomycetes | Pirellula | Immune gene | cytMnSOD | 0.833 | 0.01 |
| Planctomycetes | Pirellula | Immune gene | proPO | 0.833 | 0.01 |
| Planctomycetes | Pirellula | Immune gene | IL10 | 0.881 | 3.90E-03 |
| Planctomycetes | Pirellula | Immune gene | TNF-alpha | 0.714 | 0.047 |
| Planctomycetes | Pirellula | Immune gene | IL1-beta | 0.905 | 2.00E-03 |
| Planctomycetes | Pirellula | Immune gene | IL8 | 0.524 | 0.183 |
| Planctomycetes | SH-PL14 | Biochemical param | Energy | -0.619 | 0.102 |

| | | | | | |
|---|---|---|---|---|---|
| Planctomycetes | SH-PL14 | Hemolymph param | THC | 0.595 | 0.12 |
| Planctomycetes | SH-PL14 | Immune gene | IL17F | 0.69 | 0.058 |
| Planctomycetes | SH-PL14 | Immune gene | cytMnSOD | 0.786 | 0.021 |
| Planctomycetes | SH-PL14 | Immune gene | proPO | 0.69 | 0.058 |
| Planctomycetes | SH-PL14 | Immune gene | IL10 | 0.738 | 0.037 |
| Planctomycetes | SH-PL14 | Immune gene | TNF-alpha | 0.571 | 0.139 |
| Planctomycetes | SH-PL14 | Immune gene | IL1-beta | 0.857 | 6.50E-03 |
| Planctomycetes | SH-PL14 | Immune gene | IL8 | 0.452 | 0.26 |
| Planctomycetes | Pir4 lineage | Biochemical param | Energy | -0.714 | 0.047 |
| Planctomycetes | Pir4 lineage | Hemolymph param | THC | 0.786 | 0.021 |
| Planctomycetes | Pir4 lineage | Immune gene | IL17F | 0.595 | 0.12 |
| Planctomycetes | Pir4 lineage | Immune gene | cytMnSOD | 0.786 | 0.021 |
| Planctomycetes | Pir4 lineage | Immune gene | proPO | 0.69 | 0.058 |
| Planctomycetes | Pir4 lineage | Immune gene | IL10 | 0.738 | 0.037 |
| Planctomycetes | Pir4 lineage | Immune gene | TNF-alpha | 0.857 | 6.50E-03 |
| Planctomycetes | Pir4 lineage | Immune gene | IL1-beta | 0.857 | 6.50E-03 |
| Planctomycetes | Pir4 lineage | Immune gene | IL8 | 0.738 | 0.037 |
| Proteobacteria | Reyranella | Biochemical param | Energy | -0.714 | 0.047 |
| Proteobacteria | Reyranella | Hemolymph param | THC | 0.69 | 0.058 |
| Proteobacteria | Reyranella | Immune gene | IL17F | 0.667 | 0.071 |
| Proteobacteria | Reyranella | Immune gene | cytMnSOD | 0.762 | 0.028 |
| Proteobacteria | Reyranella | Immune gene | proPO | 0.857 | 6.50E-03 |
| Proteobacteria | Reyranella | Immune gene | IL10 | 0.833 | 0.01 |
| Proteobacteria | Reyranella | Immune gene | TNF-alpha | 0.762 | 0.028 |
| Proteobacteria | Reyranella | Immune gene | IL1-beta | 0.857 | 6.50E-03 |
| Proteobacteria | Reyranella | Immune gene | IL8 | 0.571 | 0.139 |
| Proteobacteria | Paracoccus | Biochemical param | Energy | -0.714 | 0.047 |
| Proteobacteria | Paracoccus | Hemolymph param | THC | 0.69 | 0.058 |
| Proteobacteria | Paracoccus | Immune gene | IL17F | 0.667 | 0.071 |
| Proteobacteria | Paracoccus | Immune gene | cytMnSOD | 0.762 | 0.028 |
| Proteobacteria | Paracoccus | Immune gene | proPO | 0.857 | 6.50E-03 |
| Proteobacteria | Paracoccus | Immune gene | IL10 | 0.833 | 0.01 |
| Proteobacteria | Paracoccus | Immune gene | TNF-alpha | 0.762 | 0.028 |
| Proteobacteria | Paracoccus | Immune gene | IL1-beta | 0.857 | 6.50E-03 |
| Proteobacteria | Paracoccus | Immune gene | IL8 | 0.571 | 0.139 |
| Proteobacteria | alphaI cluster | Biochemical param | Energy | -0.714 | 0.047 |
| Proteobacteria | alphaI cluster | Hemolymph param | THC | 0.786 | 0.021 |
| Proteobacteria | alphaI cluster | Immune gene | IL17F | 0.595 | 0.12 |

| | | | | | |
|---|---|---|---|---|---|
| Proteobacteria | alphaI cluster | Immune gene | cytMnSOD | 0.786 | 0.021 |
| Proteobacteria | alphaI cluster | Immune gene | proPO | 0.69 | 0.058 |
| Proteobacteria | alphaI cluster | Immune gene | IL10 | 0.738 | 0.037 |
| Proteobacteria | alphaI cluster | Immune gene | TNF-alpha | 0.857 | 6.50E-03 |
| Proteobacteria | alphaI cluster | Immune gene | IL1-beta | 0.857 | 6.50E-03 |
| Proteobacteria | alphaI cluster | Immune gene | IL8 | 0.738 | 0.037 |
| Proteobacteria | Aquabacterium | Biochemical param | Energy | -0.619 | 0.102 |
| Proteobacteria | Aquabacterium | Hemolymph param | THC | 0.595 | 0.12 |
| Proteobacteria | Aquabacterium | Immune gene | IL17F | 0.69 | 0.058 |
| Proteobacteria | Aquabacterium | Immune gene | cytMnSOD | 0.786 | 0.021 |
| Proteobacteria | Aquabacterium | Immune gene | proPO | 0.69 | 0.058 |
| Proteobacteria | Aquabacterium | Immune gene | IL10 | 0.738 | 0.037 |
| Proteobacteria | Aquabacterium | Immune gene | TNF-alpha | 0.571 | 0.139 |
| Proteobacteria | Aquabacterium | Immune gene | IL1-beta | 0.857 | 6.50E-03 |
| Proteobacteria | Aquabacterium | Immune gene | IL8 | 0.452 | 0.26 |
| Proteobacteria | Pseudomonas | Biochemical param | Energy | -0.667 | 0.071 |
| Proteobacteria | Pseudomonas | Hemolymph param | THC | 0.738 | 0.037 |
| Proteobacteria | Pseudomonas | Immune gene | IL17F | 0.643 | 0.086 |
| Proteobacteria | Pseudomonas | Immune gene | cytMnSOD | 0.69 | 0.058 |
| Proteobacteria | Pseudomonas | Immune gene | proPO | 0.595 | 0.12 |
| Proteobacteria | Pseudomonas | Immune gene | IL10 | 0.595 | 0.12 |
| Proteobacteria | Pseudomonas | Immune gene | TNF-alpha | 0.714 | 0.047 |
| Proteobacteria | Pseudomonas | Immune gene | IL1-beta | 0.81 | 0.015 |
| Proteobacteria | Pseudomonas | Immune gene | IL8 | 0.643 | 0.086 |
| Proteobacteria | Roseomonas | Biochemical param | Energy | -0.714 | 0.047 |
| Proteobacteria | Roseomonas | Hemolymph param | THC | 0.786 | 0.021 |
| Proteobacteria | Roseomonas | Immune gene | IL17F | 0.595 | 0.12 |
| Proteobacteria | Roseomonas | Immune gene | cytMnSOD | 0.786 | 0.021 |
| Proteobacteria | Roseomonas | Immune gene | proPO | 0.69 | 0.058 |
| Proteobacteria | Roseomonas | Immune gene | IL10 | 0.738 | 0.037 |
| Proteobacteria | Roseomonas | Immune gene | TNF-alpha | 0.857 | 6.50E-03 |
| Proteobacteria | Roseomonas | Immune gene | IL1-beta | 0.857 | 6.50E-03 |
| Proteobacteria | Roseomonas | Immune gene | IL8 | 0.738 | 0.037 |
| Proteobacteria | Shewanella | Biochemical param | Energy | 0.905 | 2.00E-03 |
| Proteobacteria | Shewanella | Hemolymph param | THC | -0.81 | 0.015 |
| Proteobacteria | Shewanella | Immune gene | IL17F | -0.976 | 3.30E-05 |
| Proteobacteria | Shewanella | Immune gene | cytMnSOD | -0.762 | 0.028 |
| Proteobacteria | Shewanella | Immune gene | proPO | -0.881 | 3.90E-03 |
| Proteobacteria | Shewanella | Immune gene | IL10 | -0.81 | 0.015 |

| | | | | | |
|---|---|---|---|---|---|
| Proteobacteria | Shewanella | Immune gene | TNF-alpha | -0.786 | 0.021 |
| Proteobacteria | Shewanella | Immune gene | IL1-beta | -0.643 | 0.086 |
| Proteobacteria | Shewanella | Immune gene | IL8 | -0.738 | 0.037 |
| Proteobacteria | Vibrio | Biochemical param | Energy | 0.714 | 0.047 |
| Proteobacteria | Vibrio | Hemolymph param | THC | -0.548 | 0.16 |
| Proteobacteria | Vibrio | Immune gene | IL17F | -0.738 | 0.037 |
| Proteobacteria | Vibrio | Immune gene | cytMnSOD | -0.929 | 8.60E-04 |
| Proteobacteria | Vibrio | Immune gene | proPO | -0.738 | 0.037 |
| Proteobacteria | Vibrio | Immune gene | IL10 | -0.786 | 0.021 |
| Proteobacteria | Vibrio | Immune gene | TNF-alpha | -0.667 | 0.071 |
| Proteobacteria | Vibrio | Immune gene | IL1-beta | -0.952 | 2.60E-04 |
| Proteobacteria | Vibrio | Immune gene | IL8 | -0.595 | 0.12 |
| Tenericutes | Candidatus Hepatoplasma | Biochemical param | Energy | -0.929 | 8.60E-04 |
| Tenericutes | Candidatus Hepatoplasma | Hemolymph param | THC | 0.762 | 0.028 |
| Tenericutes | Candidatus Hepatoplasma | Immune gene | IL17F | 0.881 | 3.90E-03 |
| Tenericutes | Candidatus Hepatoplasma | Immune gene | cytMnSOD | 0.881 | 3.90E-03 |
| Tenericutes | Candidatus Hepatoplasma | Immune gene | proPO | 0.81 | 0.015 |
| Tenericutes | Candidatus Hepatoplasma | Immune gene | IL10 | 0.762 | 0.028 |
| Tenericutes | Candidatus Hepatoplasma | Immune gene | TNF-alpha | 0.905 | 2.00E-03 |
| Tenericutes | Candidatus Hepatoplasma | Immune gene | IL1-beta | 0.81 | 0.015 |
| Tenericutes | Candidatus Hepatoplasma | Immune gene | IL8 | 0.905 | 2.00E-03 |
| Tenericutes | Candidatus Bacilloplasma | Biochemical param | Energy | 0.714 | 0.047 |
| Tenericutes | Candidatus Bacilloplasma | Hemolymph param | THC | -0.738 | 0.037 |
| Tenericutes | Candidatus Bacilloplasma | Immune gene | IL17F | -0.738 | 0.037 |
| Tenericutes | Candidatus Bacilloplasma | Immune gene | cytMnSOD | -0.929 | 8.60E-04 |
| Tenericutes | Candidatus Bacilloplasma | Immune gene | proPO | -0.833 | 0.01 |
| Tenericutes | Candidatus Bacilloplasma | Immune gene | IL10 | -0.976 | 3.30E-05 |
| Tenericutes | Candidatus Bacilloplasma | Immune gene | TNF-alpha | -0.762 | 0.028 |
| Tenericutes | Candidatus Bacilloplasma | Immune gene | IL1-beta | -0.857 | 6.50E-03 |

| | | | | | |
|---|---|---|---|---|---|
| Tenericutes | Candidatus Bacilloplasma | Immune gene | IL8 | -0.643 | 0.086 |
| Verrucomicrobia | Terrimicrobium | Biochemical param | Energy | -0.714 | 0.047 |
| Verrucomicrobia | Terrimicrobium | Hemolymph param | THC | 0.786 | 0.021 |
| Verrucomicrobia | Terrimicrobium | Immune gene | IL17F | 0.595 | 0.12 |
| Verrucomicrobia | Terrimicrobium | Immune gene | cytMnSOD | 0.738 | 0.037 |
| Verrucomicrobia | Terrimicrobium | Immune gene | proPO | 0.738 | 0.037 |
| Verrucomicrobia | Terrimicrobium | Immune gene | IL10 | 0.738 | 0.037 |
| Verrucomicrobia | Terrimicrobium | Immune gene | TNF-alpha | 0.857 | 6.50E-03 |
| Verrucomicrobia | Terrimicrobium | Immune gene | IL1-beta | 0.857 | 6.50E-03 |
| Verrucomicrobia | Terrimicrobium | Immune gene | IL8 | 0.714 | 0.047 |
| Verrucomicrobia | Luteolibacter | Biochemical param | Energy | -0.714 | 0.047 |
| Verrucomicrobia | Luteolibacter | Hemolymph param | THC | 0.69 | 0.058 |
| Verrucomicrobia | Luteolibacter | Immune gene | IL17F | 0.667 | 0.071 |
| Verrucomicrobia | Luteolibacter | Immune gene | cytMnSOD | 0.762 | 0.028 |
| Verrucomicrobia | Luteolibacter | Immune gene | proPO | 0.857 | 6.50E-03 |
| Verrucomicrobia | Luteolibacter | Immune gene | IL10 | 0.833 | 0.01 |
| Verrucomicrobia | Luteolibacter | Immune gene | TNF-alpha | 0.762 | 0.028 |
| Verrucomicrobia | Luteolibacter | Immune gene | IL1-beta | 0.857 | 6.50E-03 |
| Verrucomicrobia | Luteolibacter | Immune gene | IL8 | 0.571 | 0.139 |
| Verrucomicrobia | Lactobacillus | Biochemical param | Energy | -0.667 | 0.071 |
| Verrucomicrobia | Lactobacillus | Hemolymph param | THC | 0.643 | 0.086 |
| Verrucomicrobia | Lactobacillus | Immune gene | IL17F | 0.643 | 0.086 |
| Verrucomicrobia | Lactobacillus | Immune gene | cytMnSOD | 0.833 | 0.01 |
| Verrucomicrobia | Lactobacillus | Immune gene | proPO | 0.833 | 0.01 |
| Verrucomicrobia | Lactobacillus | Immune gene | IL10 | 0.881 | 3.90E-03 |
| Verrucomicrobia | Lactobacillus | Immune gene | TNF-alpha | 0.714 | 0.047 |
| Verrucomicrobia | Lactobacillus | Immune gene | IL1-beta | 0.905 | 2.00E-03 |
| Verrucomicrobia | Lactobacillus | Immune gene | IL8 | 0.524 | 0.183 |

**Supplementary Table 3.** Ingredients and proximate composition of the experimental diet

| Ingredients (g/100g)[⁋] | Basal diet |
|---|---|
| Fishmeal (Anchovy) | 41.00 |
| Soya bean meal | 10.00 |
| Wheat flour | 37.00 |
| Corn starch | 4.80 |
| Cod liver oil | 4.20 |
| $CaCO_3$ | 0.02 |
| Vitamin premix | 0.23 |
| Vitamin C | 0.05 |
| Cholesterol | 0.50 |
| Lecithin-Soy | 1.00 |
| Betacaine | 1.20 |
| *Nutrient composition (% dry weight)* | |
| Crude protein | 29.85 |
| Crude lipid | 7.42 |
| Gross energy (MJ/kg) | 18.86 |
| *Lactobacillus acidophilus* (CFU/mL) | $1.01 \times 10^9$ |
| *Lactobacillus plantarum* (CFU/mL) | $1.05 \times 10^9$ |

Note: [⁋]All ingredients were procured and feeds were formulated by Glen Forest Specialty Feeds, Western Australia.

**Supplementary Table 4.** Primers used for gene expression analysis

| Primer name | Forward sequence (5′-3′) | Reverse sequence (5′-3′) | Reference |
|---|---|---|---|
| proPO | GCCAAGGATCTTTGTGATGTCTT | CGGCCGGCCAGTTCTAT | [66] |
| cytMnSOD | AGGTCGAGCAAGCAGGTGTAG | GTGGGAATAAACTGCAGCAATCT | [66] |
| PcCTSL | CGGATCACTGGAGGGTCAAACACTT | GCAATTTTCATCCTCGGCATCAT | [68] |
| IL1β | GTTTACCTGAACATGTCGGC | AGGGTGCTGATGTTCAGCCC | [7] |
| IL-8 | CTATTGTGGTGTTCCTGA | TCTTCACCCAGGGAGCTTC | [7] |
| IL-10 | CAGTGCAGAAGAGTCGACTGCAAG | CGCTTGAGATCCTGAAATATA | [7] |
| IL-17F | GTCTCTGTCACCGTGGAC | TGGGCCTCACACAGGTACA | [7] |
| TNF-α | TGGAGGGGTATGCGATGACACCTG | TGAGGCCTTTCTCTCAGCGACAGC | [7] |
| vg | CCAGAAGACGCCACAAGAA | CAGAAGGCATCAGCCAATC | [67] |
| pcna | AGAGGCGGACTGAAGAGG | TTGATGGCATCCAGCACT | [67] |
| β-actin | TTGAGCAGGAGATGGGAACCG | AGAGCCTCAGGGCAACGGAAA | [67] |
